# Supplementary material for: A Pan-Transcriptome Analysis Indicates Efficient Downregulation of the FIB Genes Plays a Critical Role in the Response of Alfalfa to Cold Stress
Source: Plants (Basel). 2022 Nov 17;11(22):3148. doi: 10.3390/plants11223148 (PMC9692835; doi:10.3390/plants11223148)
Supplement: Supplementary file 1 [file plants-11-03148-s001.zip › Table S1.pdf]

Table S1 Summary of eight alfalfa varieties

Index	Variety	FD	Group	Source
A	Algonquin	2	FDT	Barenbrug China Ltd. Com.
B	WL168HQ	2	FDT	Barenbrug China Ltd. Com.
C	WL319HQ	2.8	FDT	Barenbrug China Ltd. Com.
D	Bara310SC	3	FDT	Barenbrug China Ltd. Com.
E	WL440HQ	6	NDT	Barenbrug China Ltd. Com.
F	Eureka+	8	NDT	Barenbrug China Ltd. Com.
G	WL525HQ	8.2	NDT	Barenbrug China Ltd. Com.
H	WL903HQ	9.5	NDT	Barenbrug China Ltd. Com.

Note, all alfalfa varieties were collected from Barenbrug China Ltd. Com., and the FD (fall dormancy) values were collected from instruction of each variety. FDT, fall dormant type; NDT, non-dormant type.
