# Supplementary material for: A Pan-Transcriptome Analysis Indicates Efficient Downregulation of the FIB Genes Plays a Critical Role in the Response of Alfalfa to Cold Stress
Source: Plants (Basel). 2022 Nov 17;11(22):3148. doi: 10.3390/plants11223148 (PMC9692835; doi:10.3390/plants11223148)
Supplement: Supplementary file 1 [file plants-11-03148-s001.zip › Table S2.pdf]

Table S2 Summary of RNA-Seq reads

Variety	Repeat	Clean Reads	Q20(%)	GC(%)
Algonquin	A1	26,947,872	96.91%	42.65%
	A2	27,177,370	96.88%	42.34%
	A3	27,966,244	97.45%	42.25%
WL168HQ	B1	27,929,110	97.11%	41.92%
	B2	27,196,292	96.92%	42.59%
	B3	27,272,718	97.07%	41.98%
WL319HQ	C1	27,197,458	96.62%	42.48%
	C2	27,002,808	96.77%	42.18%
	C3	27,203,324	96.85%	42.76%
Bara310SC	D1	27,219,556	96.78%	42.34%
	D2	27,144,282	97.03%	42.17%
	D3	27,033,292	97.14%	41.99%
WL440HQ	E1	26,917,608	97.33%	42.34%
	E2	28,085,064	97.47%	42.29%
	E3	27,942,522	97.41%	42.92%
Eureka+	F1	27,234,476	97.43%	42.16%
	F2	27,700,458	97.46%	42.59%
	F3	28,119,812	97.44%	42.32%
WL525HQ	G1	28,062,412	97.36%	42.65%
	G2	27,895,558	97.36%	42.41%
	G3	27,701,320	97.52%	41.83%
WL903HQ	H1	27,968,900	97.55%	41.74%
	H2	28,200,830	97.52%	41.61%
	H3	27,579,924	97.55%	42.22%

Total	660,699,210
