# Supplementary material for: A Pan-Transcriptome Analysis Indicates Efficient Downregulation of the FIB Genes Plays a Critical Role in the Response of Alfalfa to Cold Stress
Source: Plants (Basel). 2022 Nov 17;11(22):3148. doi: 10.3390/plants11223148 (PMC9692835; doi:10.3390/plants11223148)
Supplement: Supplementary file 1 [file plants-11-03148-s001.zip › Table S3.pdf]

Table S3 Summary of RNA-seq reads mapping to different alfalfa gene sequences

Variety	Repeat	UniGenes	*Medicago truncatula*	Alfalfa cv. Xingjiang Daye	Alfalfa cv. ZhongmuNo.1
	A1	78.48	76.54	72.67	67.99
Algonquin	A2	81.69	77.14	73.10	67.20
	A3	81.15	78.11	73.68	68.24
	B1	79.98	75.64	70.51	65.78
WL168HQ	B2	79.07	73.93	69.19	64.13
	B3	79.09	72.36	67.39	63.46
	C1	79.33	75.96	71.59	67.26
WL319HQ	C2	80.25	76.44	72.31	66.72
	C3	78.49	76.12	73.52	68.55
	D1	80.06	76.31	74.09	68.38
Bara310SC	D2	80.46	76.61	73.35	68.19
	D3	81.04	75.36	70.12	65.29
	E1	80.43	74.48	70.57	66.17
WL440HQ	E2	80.70	75.83	72.53	67.51
	E3	78.02	75.89	74.69	70.15
	F1	80.56	75.55	71.50	65.58
Eureka+	F2	78.48	75.45	72.67	68.67
	F3	79.27	75.98	72.85	67.86
	G1	79.42	76.73	74.76	69.79
WL525HQ	G2	79.76	77.00	74.85	69.68
	G3	80.65	75.04	71.41	65.77
	H1	79.02	75.26	71.13	66.10
WL903HQ	H2	80.99	76.95	71.83	66.00
	H3	80.20	76.64	72.95	68.06
Mean		79.86	75.89	72.22	67.19
STD		0.99	1.17	1.82	1.73

Note, Unigenes were obtained in our work, Medicago transcripts were downloaded from Medicago genome from Phytozome site, Xingjiang Daye genes (CDS) were downloaded from alfalfa Xingjiang Daye genome, as ref described., while ZhongmuNo.1 genes (CDS) were downloaded from alfalfa variety ZhongmuNo.1 genome, as ref described.
