# Supplementary material for: A Pan-Transcriptome Analysis Indicates Efficient Downregulation of the FIB Genes Plays a Critical Role in the Response of Alfalfa to Cold Stress
Source: Plants (Basel). 2022 Nov 17;11(22):3148. doi: 10.3390/plants11223148 (PMC9692835; doi:10.3390/plants11223148)
Supplement: Supplementary file 1 [file plants-11-03148-s001.zip › Table S4.pdf]

Table S4 Gene annotation of key genes involving in ribosome biogenesis process

Transcript	Homolog	Gene Description	Note
MsaG03248	AT4G25630	FIB2	Hub Gene
MsaG39376	AT5G52470	ATFBR1, ATFIB1, FBR1, FIB1, FIBRILLARIN 1, SKIP7, SKP1/ASK1-INTERACTING PROTEIN	
MsaG07867	AT3G09200	Ribosomal protein L10 family protein	
MsaG17011	AT5G16750	TOZ	
MsaG19217	AT5G66540	U3 small nucleolar ribonucleoprotein	
MsaG23794	AT4G25730	ATTRM7B, TRM7B, TRNA METHYLTRANSFERASE 7B	
MsaG36284	AT2G40360	ARABIDOPSIS THALIANA PESCADILLO ORTHOLOG1, ATPEIP1, ATPEP1, BLOCK OF CELL PROLIFERATION 1, BOP1	
MsaG01295	AT1G25260	RDP	
MsaG62257	AT1G06720	P-loop containing nucleoside triphosphate hydrolases superfamily protein	
MsaG72031	Medtr0072s0050	AT1G03530/ATNAF1, NAF1, NUCLEAR ASSEMBLY FACTOR 1	
