# Supplementary material for: A Pan-Transcriptome Analysis Indicates Efficient Downregulation of the FIB Genes Plays a Critical Role in the Response of Alfalfa to Cold Stress
Source: Plants (Basel). 2022 Nov 17;11(22):3148. doi: 10.3390/plants11223148 (PMC9692835; doi:10.3390/plants11223148)
Supplement: Supplementary file 1 [file plants-11-03148-s001.zip › Table S5.pdf]

Table S5 GO enrichment analysis of genes from important modules

Module	GO ID	Description
**MElightyellow**	GO:0016469(8.5e-05)	proton-transporting two-sector ATPase complex
**MEbrown4**	GO:0042254(2.1e-10)	ribosome biogenesis
**MEtan**	GO:0003700(5.0e-07)	DNA-binding transcription factor activity
**MElightpink4**	GO:0003700(0.0106)	DNA-binding transcription factor activity
**MEcoral1**	GO:0016491(6.5e-05)	oxidoreductase activity
**MEgreen**	GO:0016491(0.00312)	oxidoreductase activity
**MEdarkorange**	GO:0015979(1.3e-11)<br>GO:0016717(8.2e-05)	photosynthesis<br>oxidoreductase activity, acting on paired donors, with oxidation of a pair of donors resulting in the reduction of molecular oxygen to two molecules of water
**MEbisque4**	GO:0016787(1.1e-08)	hydrolase activity
**MEyellow**	GO:0009055(1.3e-05)	electron transfer activity
**MEthistle2**	GO:0009117(0.0047)	nucleotide metabolic process
