# Supplementary figures and images for: A Pan-Transcriptome Analysis Indicates Efficient Downregulation of the FIB Genes Plays a Critical Role in the Response of Alfalfa to Cold Stress
Source: Plants (Basel). 2022 Nov 17;11(22):3148. doi: 10.3390/plants11223148 (PMC9692835; doi:10.3390/plants11223148)

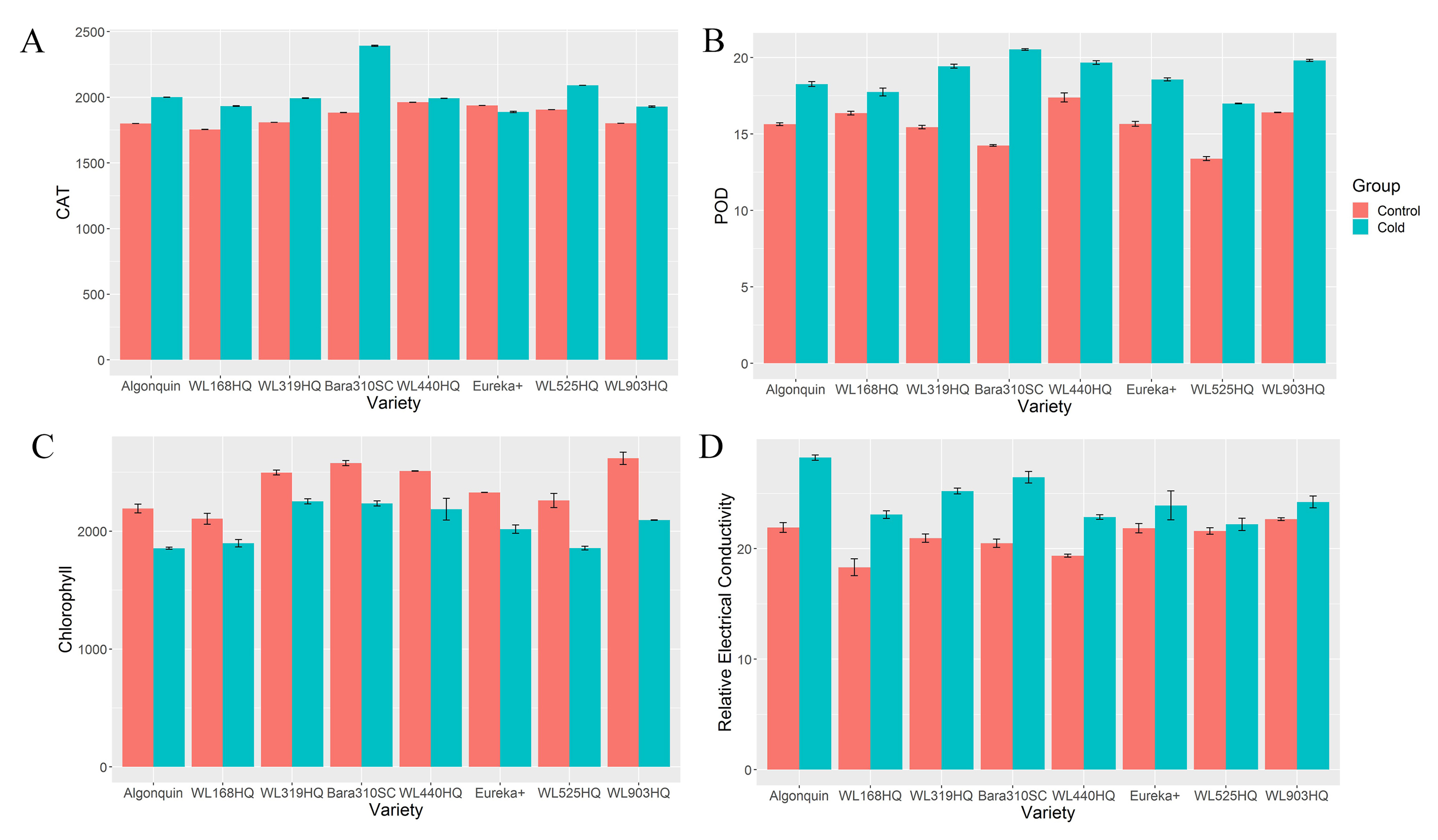

Supplement: Supplementary file 1 [file plants-11-03148-s001.zip › Figure S1.jpg]

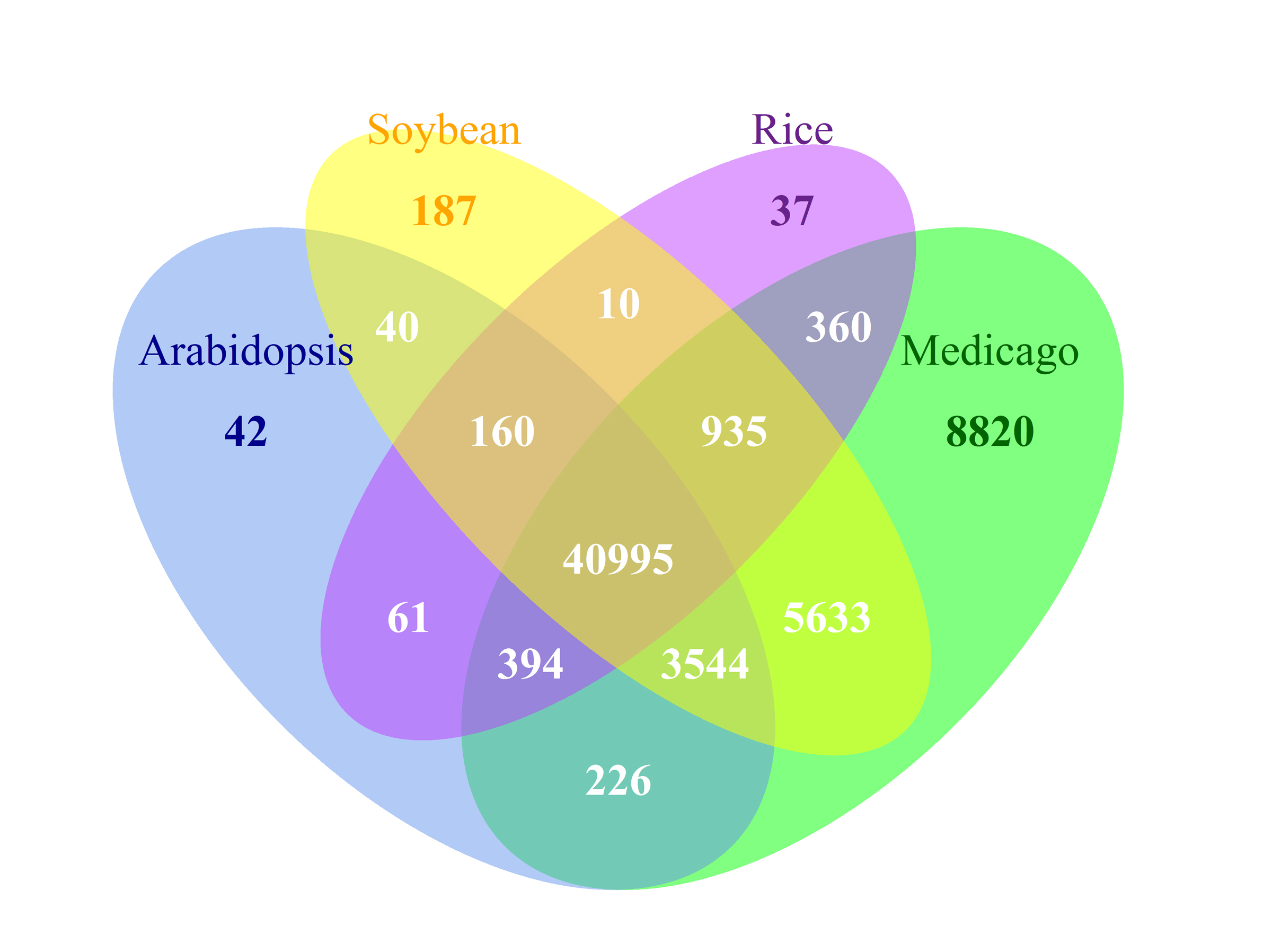

Supplement: Supplementary file 1 [file plants-11-03148-s001.zip › Figure S2 Venn_MsaPEP.jpg]

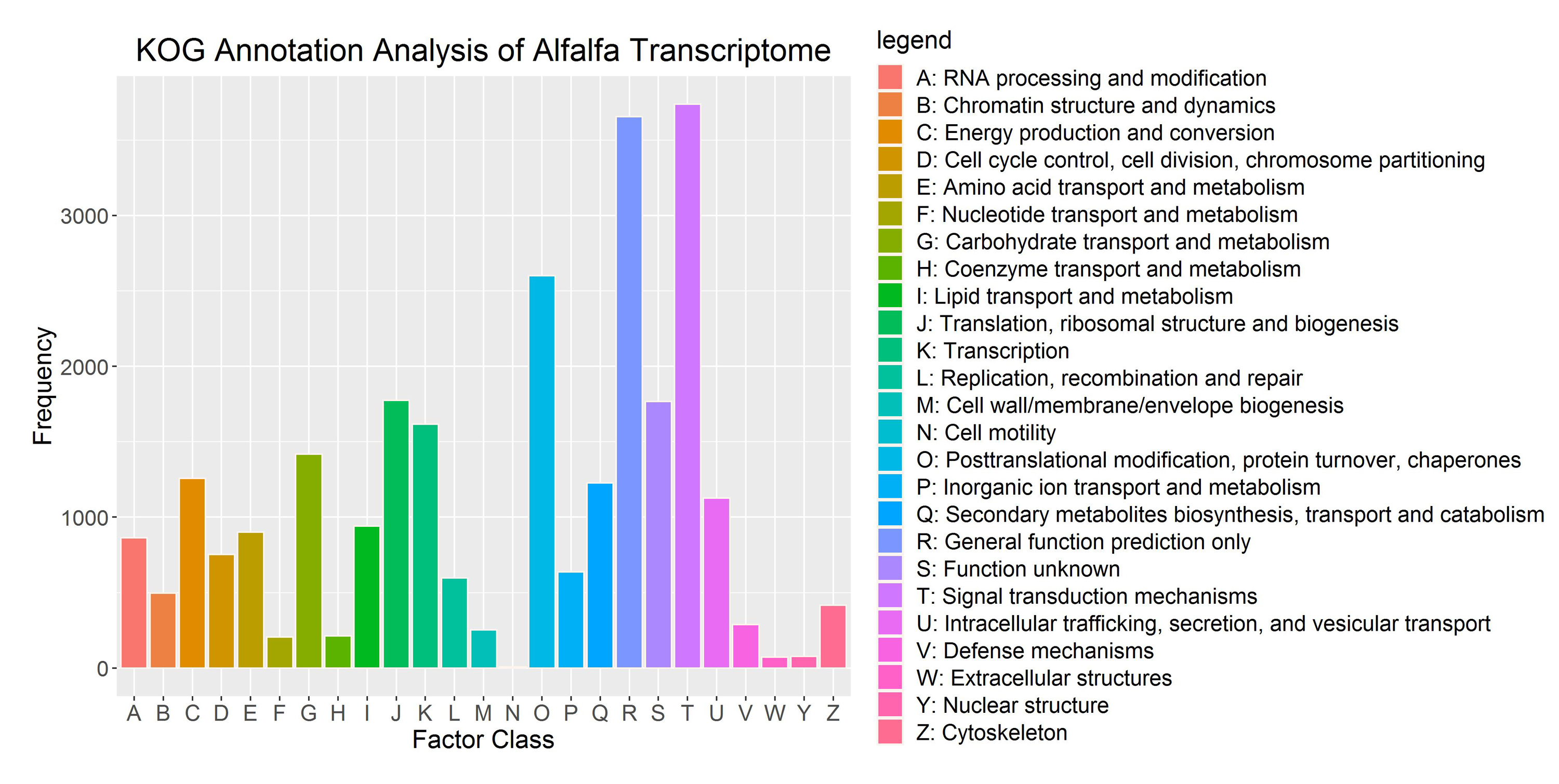

Supplement: Supplementary file 1 [file plants-11-03148-s001.zip › Figure S3_Alfalfa_KOG_New.jpg]

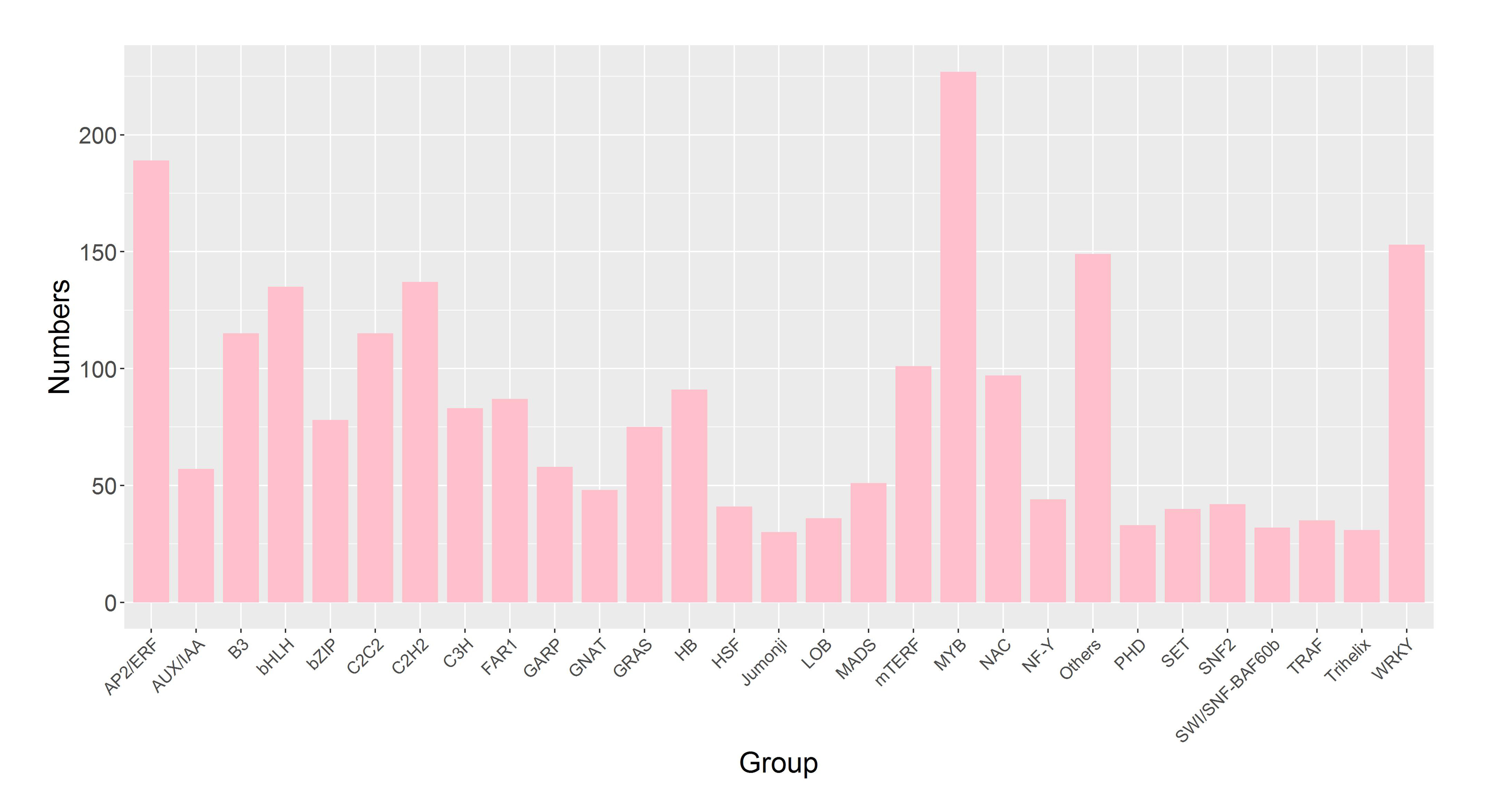

Supplement: Supplementary file 1 [file plants-11-03148-s001.zip › Figure S4_AlfalfaANNT_TF_New.jpg]

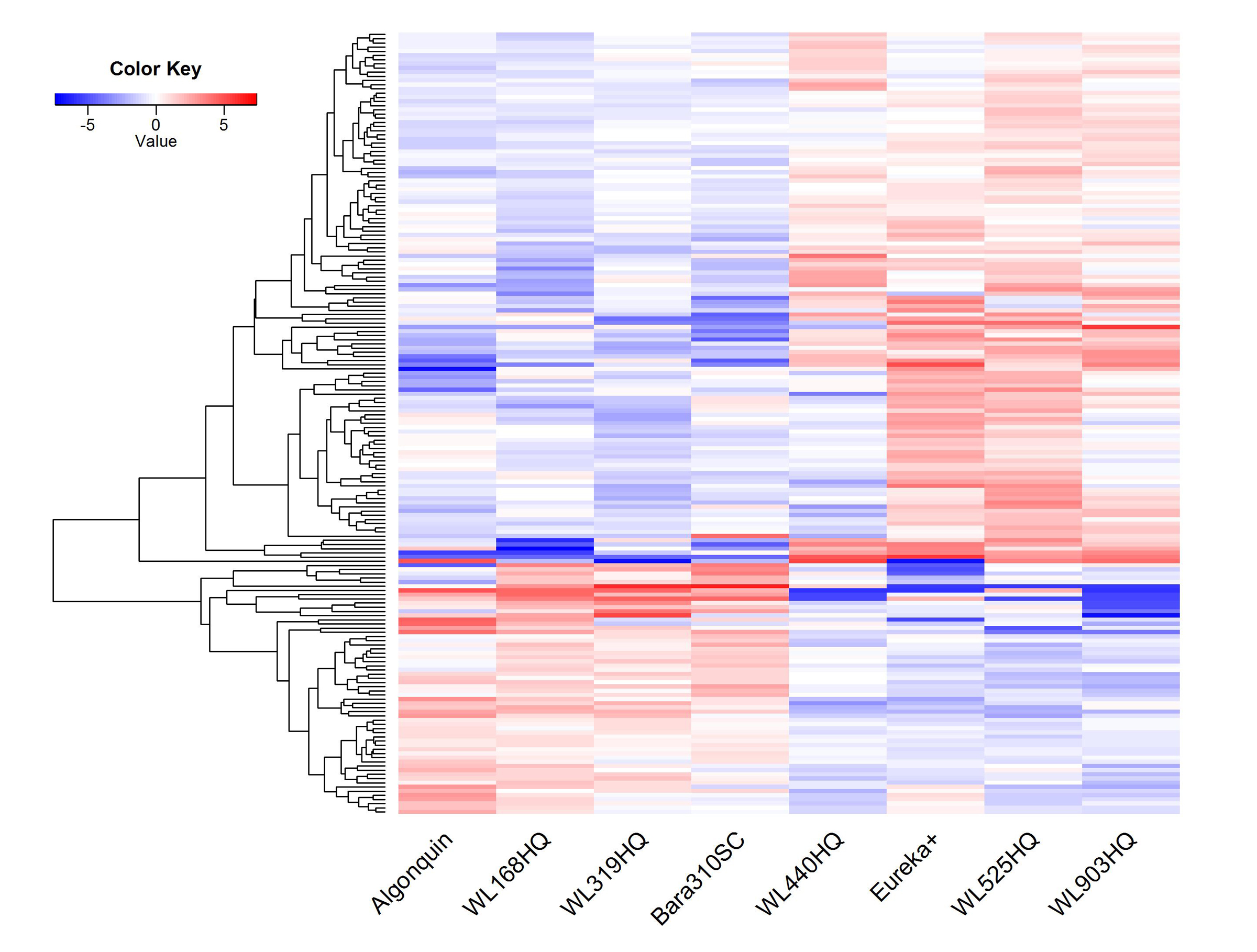

Supplement: Supplementary file 1 [file plants-11-03148-s001.zip › Figure S5 MGO0016491_cluster.jpg]

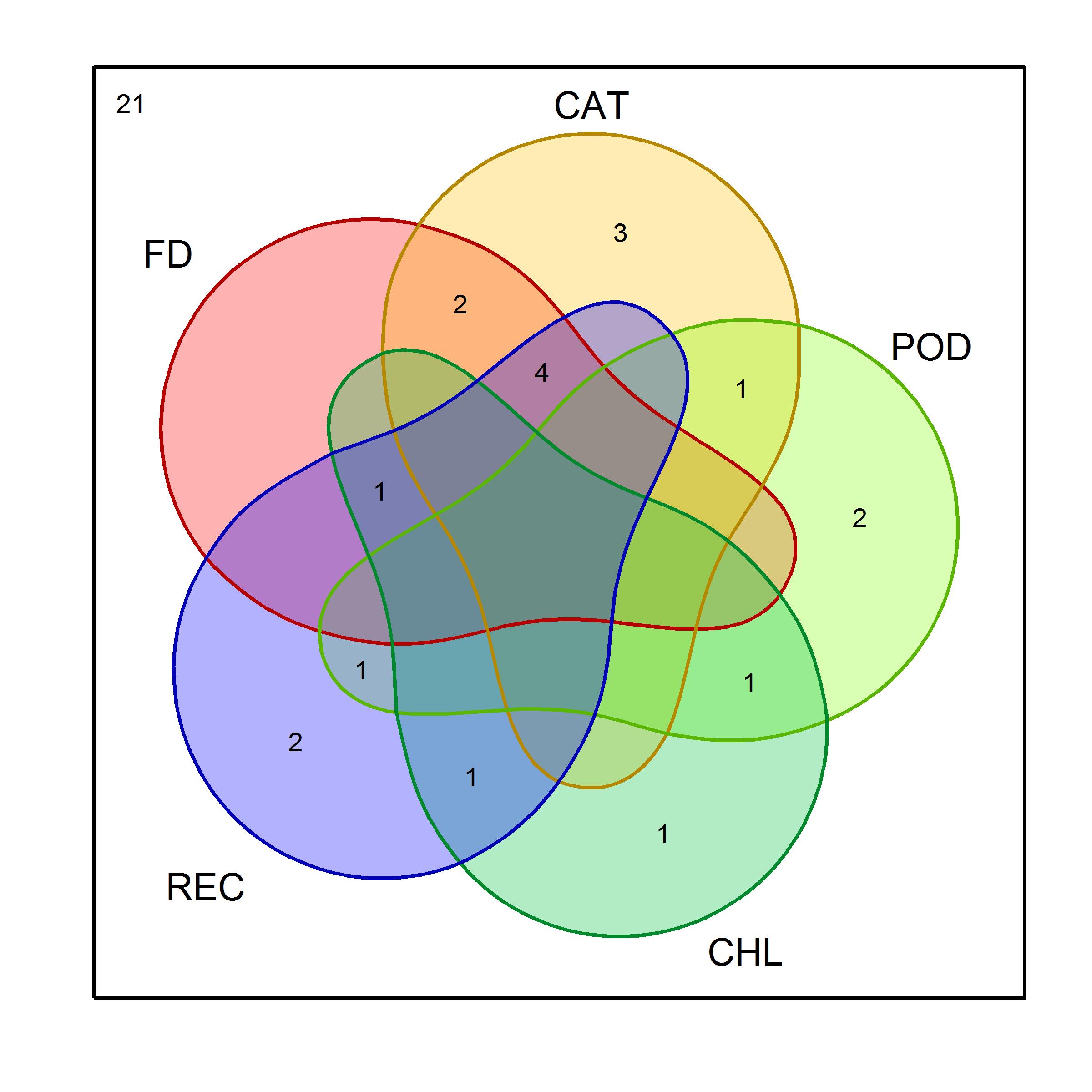

Supplement: Supplementary file 1 [file plants-11-03148-s001.zip › Figure S6 MsaMDVenn.jpeg]

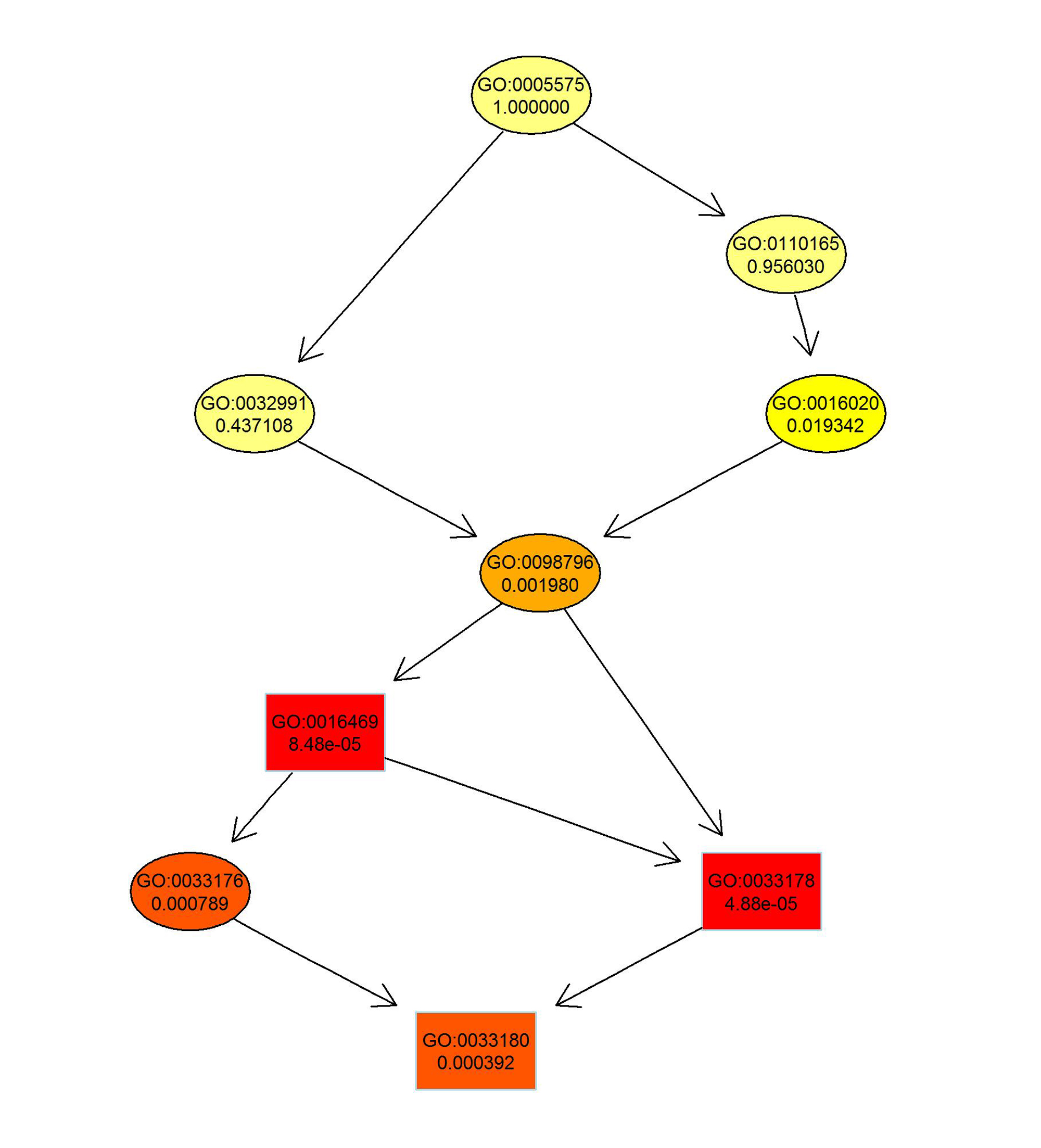

Supplement: Supplementary file 1 [file plants-11-03148-s001.zip › Figure S7_MElightyellowGON.jpg]

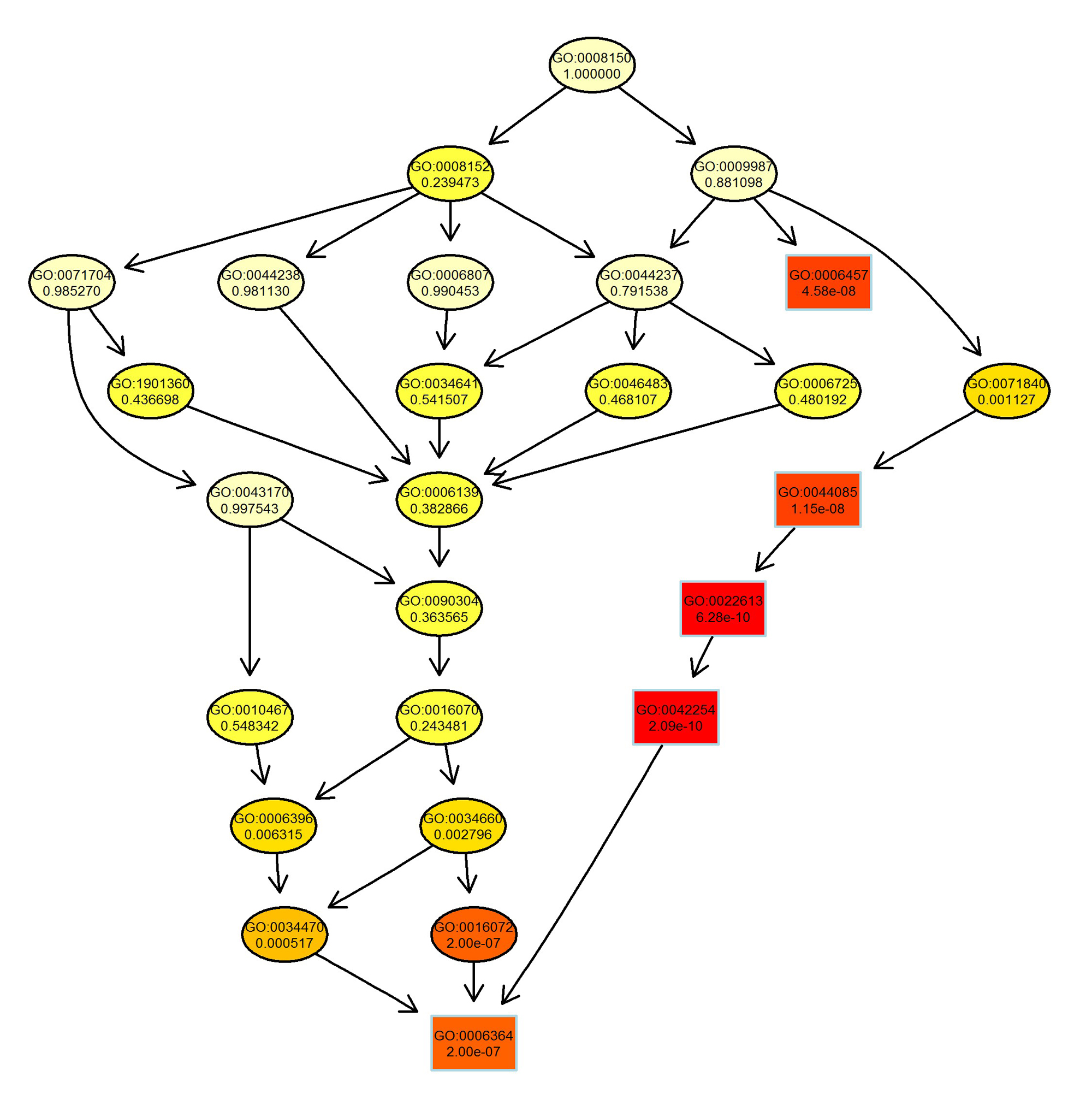

Supplement: Supplementary file 1 [file plants-11-03148-s001.zip › Figure S8_MEbrown4GON.jpg]

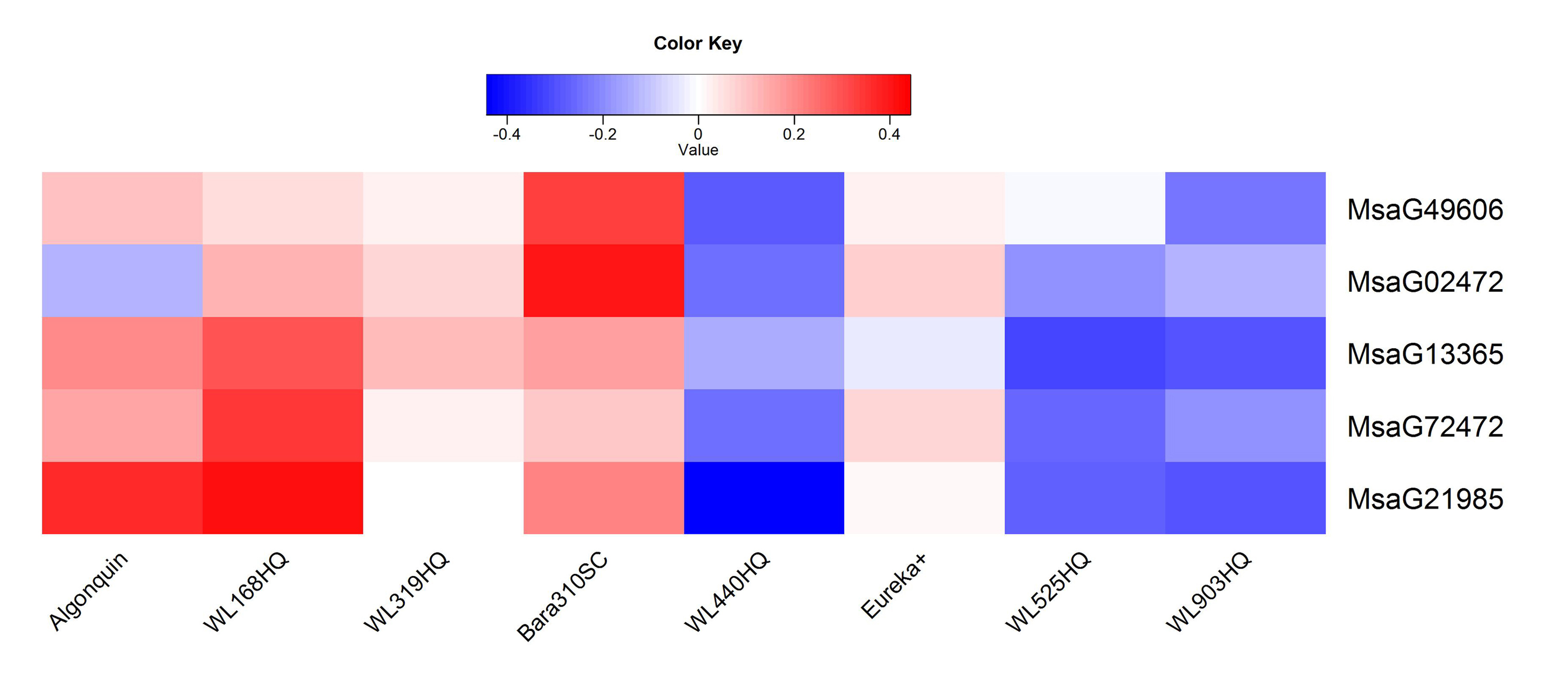

Supplement: Supplementary file 1 [file plants-11-03148-s001.zip › Figure S9 MElyATP_cluster.jpg]
